# Supplementary material for: Identification of Antiprotozoal Compounds from Buxus sempervirens L. by PLS-Prediction
Source: Molecules. 2021 Oct 13;26(20):6181. doi: 10.3390/molecules26206181 (PMC8537364; doi:10.3390/molecules26206181)
Supplement: Supplementary file 1 [file molecules-26-06181-s001.zip › molecules-1397378-supplementary.pdf]

molecules

MDPI

Supplementary Materials

Identification of Antiprotozoal Compounds from *Buxus sempervirens* L. by PLS-Prediction

Lara U. Szabó [1], Marcel Kaiser [2,3], Pascal Mäser [2,3] and Thomas J. Schmidt [1,*]

[1] Institute of Pharmaceutical Biology and Phytochemistry (IPBP), University of Münster, PharmaCampus Corrensstraße 48, D-48149 Münster, Germany; lszabo@uni-muenster.de
[2] Swiss Tropical and Public Health Institute (Swiss TPH), Socinstrasse 57, CH-4051 Basel, Switzerland; marcel.kaiser@unibas.ch (M.K.); pascal.maeser@swisstph.ch (P.M.)
[3] University of Basel, Petersplatz 1, CH-4003 Basel, Switzerland
* Correspondence: thomschm@uni-muenster.de; Tel.: +49-251-83-33378

Citation: Szabó, L.U.; Kaiser, M.; Mäser, P.; Schmidt, T.J. Identification of Antiprotozoal Compounds from *Buxus sempervirens* L. by PLS-Prediction. *Molecules* **2021**, *26*, 6181. https://doi.org/10.3390/molecules26206181

Academic Editor(s): Vera Muccilli, Monica Scognamiglio and Julien Wist

Received: 11 September 2021
Accepted: 8 October 2021
Published: 13 October 2021

Publisher's Note: MDPI stays neutral with regard to jurisdictional claims in published maps and institutional affiliations.

Copyright: © 2021 by the authors. Submitted for possible open access publication under the terms and conditions of the Creative Commons Attribution (CC BY) license (https://creativecommons.org/licenses/by/4.0/).

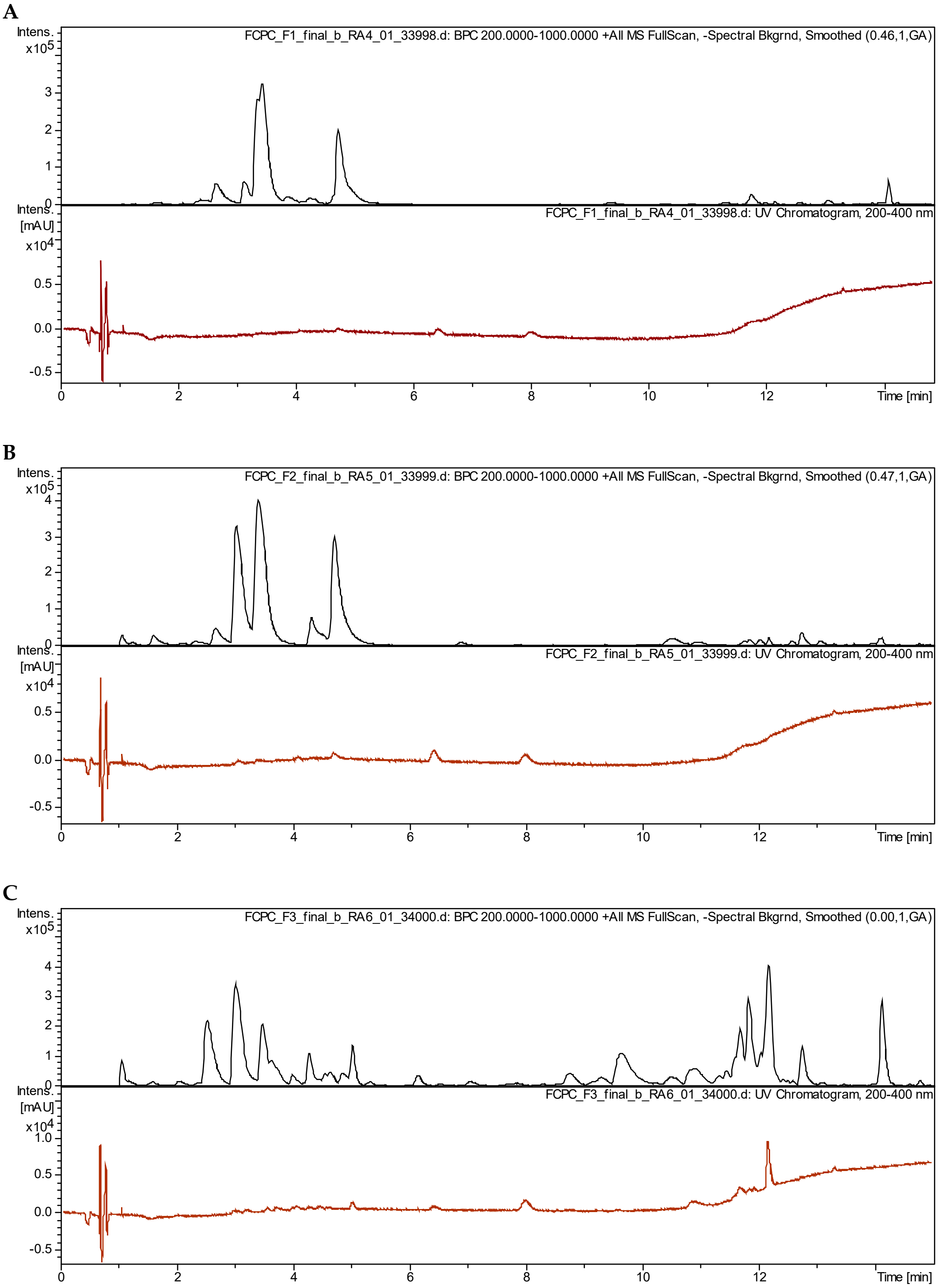
A
Intens.
x10^5
FCPC_F1_final_b_RA4_01_33998.d: BPC 200.0000-1000.0000 +All MS FullScan, -Spectral Bkgrnd, Smoothed (0.46,1,GA)
Intens.
[mAU]
x10^4
FCPC_F1_final_b_RA4_01_33998.d: UV Chromatogram, 200-400 nm
Time [min]
B
Intens.
x10^5
FCPC_F2_final_b_RA5_01_33999.d: BPC 200.0000-1000.0000 +All MS FullScan, -Spectral Bkgrnd, Smoothed (0.47,1,GA)
Intens.
[mAU]
x10^4
FCPC_F2_final_b_RA5_01_33999.d: UV Chromatogram, 200-400 nm
Time [min]
C
Intens.
x10^5
FCPC_F3_final_b_RA6_01_34000.d: BPC 200.0000-1000.0000 +All MS FullScan, -Spectral Bkgrnd, Smoothed (0.00,1,GA)
Intens.
[mAU]
x10^4
FCPC_F3_final_b_RA6_01_34000.d: UV Chromatogram, 200-400 nm
Time [min]

D

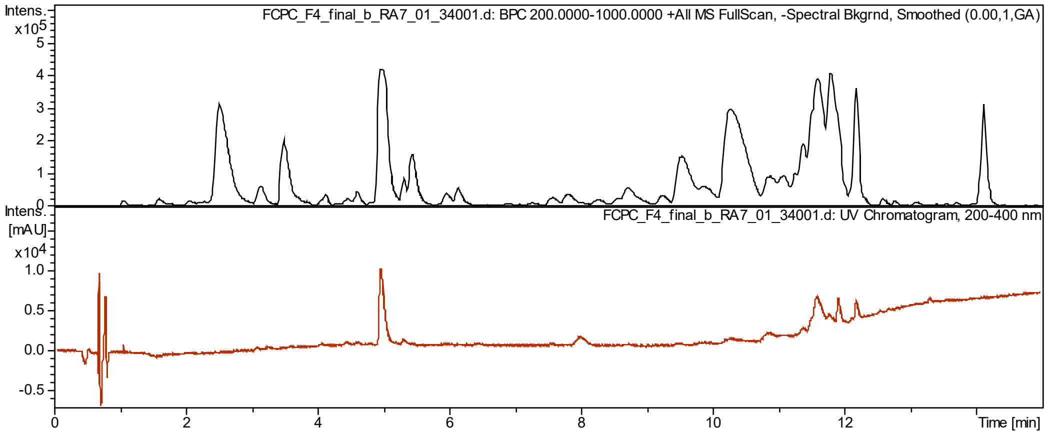
Intens.
x10^5
FCPC_F4_final_b_RA7_01_34001.d: BPC 200.0000-1000.0000 +All MS FullScan, -Spectral Bkgrnd, Smoothed (0.00,1,GA)
Intens.
[mAU]
x10^4
FCPC_F4_final_b_RA7_01_34001.d: UV Chromatogram, 200-400 nm
Time [min]

E

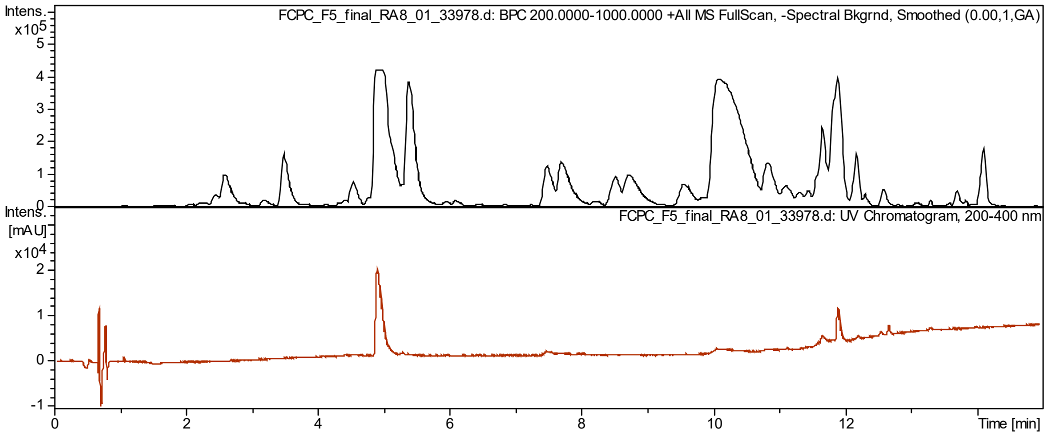
Intens.
x10^5
FCPC_F5_final_RA8_01_33978.d: BPC 200.0000-1000.0000 +All MS FullScan, -Spectral Bkgrnd, Smoothed (0.00,1,GA)
Intens.
[mAU]
x10^4
FCPC_F5_final_RA8_01_33978.d: UV Chromatogram, 200-400 nm
Time [min]

F

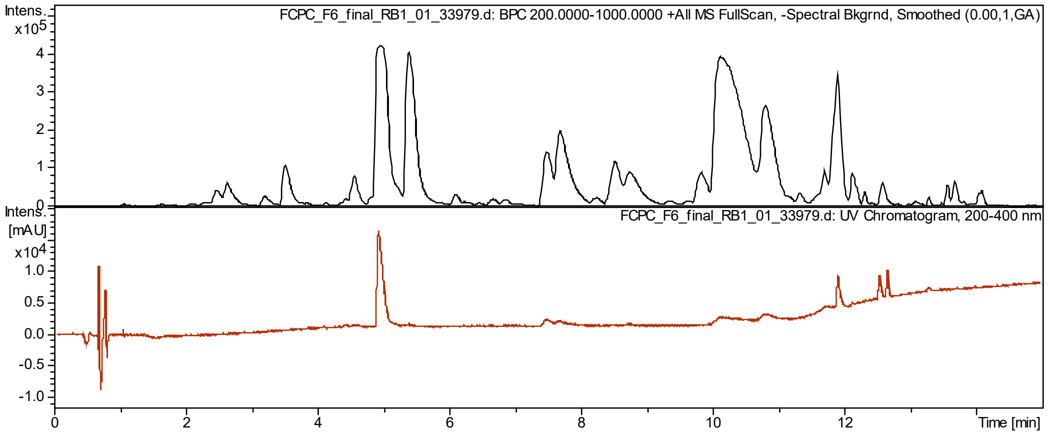
Intens.
x10^5
FCPC_F6_final_RB1_01_33979.d: BPC 200.0000-1000.0000 +All MS FullScan, -Spectral Bkgrnd, Smoothed (0.00,1,GA)
Intens.
[mAU]
x10^4
FCPC_F6_final_RB1_01_33979.d: UV Chromatogram, 200-400 nm
Time [min]

G

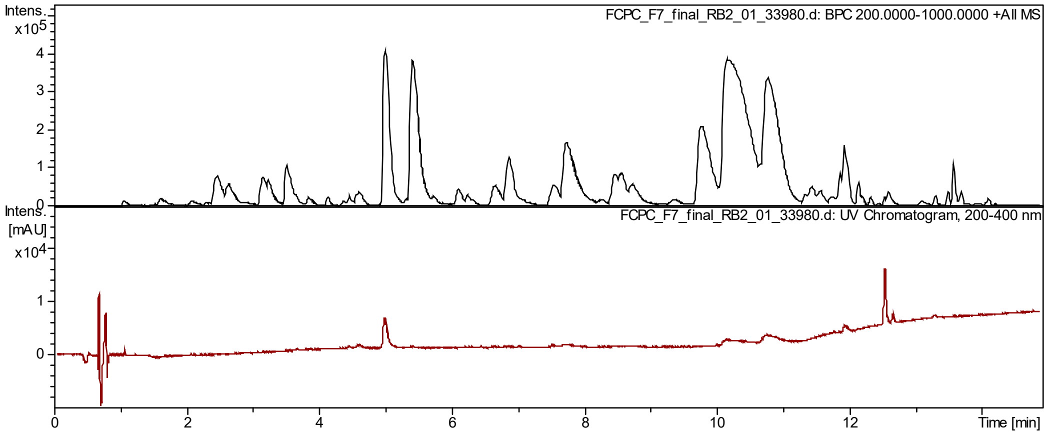

Intens.
x10^5
FCPC_F7_final_RB2_01_33980.d: BPC 200.0000-1000.0000 +All MS
Intens.
[mAU]
x10^4
FCPC_F7_final_RB2_01_33980.d: UV Chromatogram, 200-400 nm
Time [min]

H

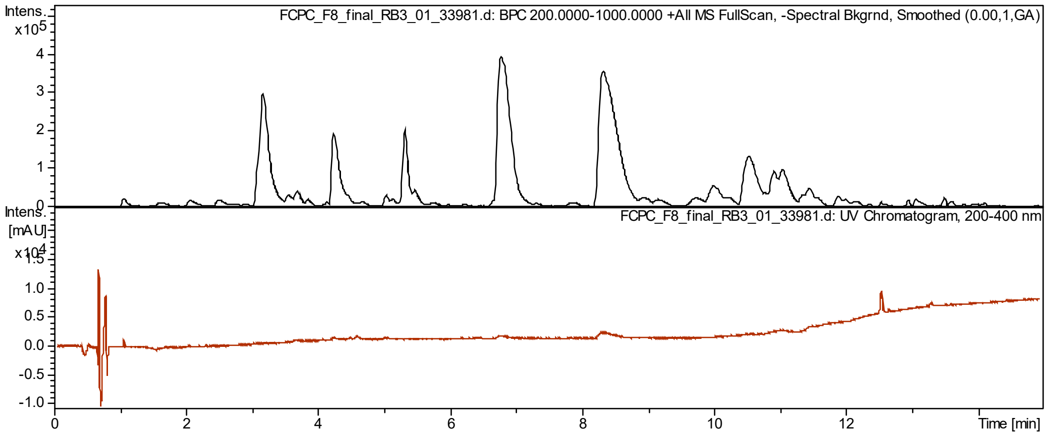

Intens.
x10^5
FCPC_F8_final_RB3_01_33981.d: BPC 200.0000-1000.0000 +All MS FullScan, -Spectral Bkgrnd, Smoothed (0.00,1,GA)
Intens.
[mAU]
x10^4
FCPC_F8_final_RB3_01_33981.d: UV Chromatogram, 200-400 nm
Time [min]

I

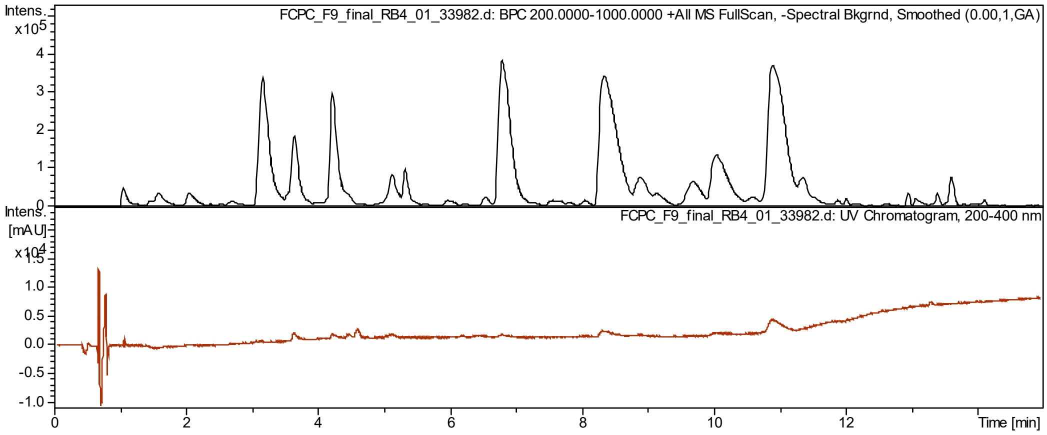

Intens.
x10^5
FCPC_F9_final_RB4_01_33982.d: BPC 200.0000-1000.0000 +All MS FullScan, -Spectral Bkgrnd, Smoothed (0.00,1,GA)
Intens.
[mAU]
x10^4
FCPC_F9_final_RB4_01_33982.d: UV Chromatogram, 200-400 nm
Time [min]

J

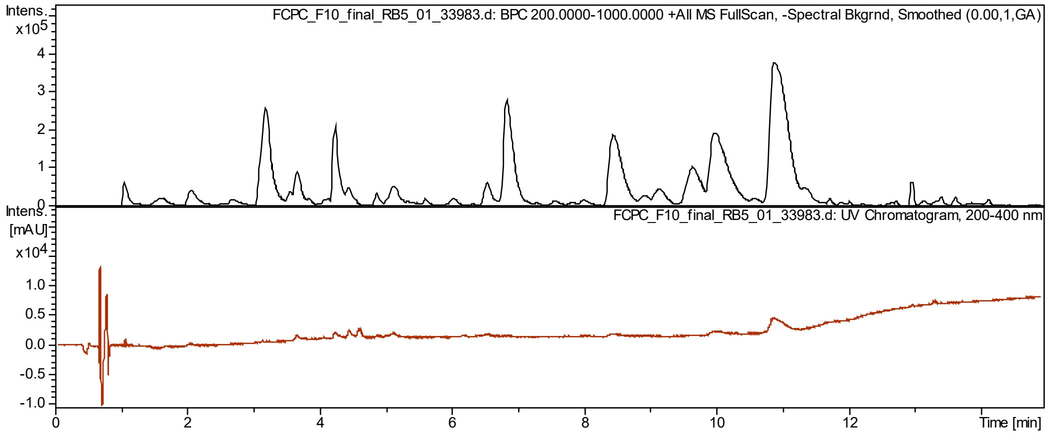
Intens.
x10^5
FCPC_F10_final_RB5_01_33983.d: BPC 200.0000-1000.0000 +All MS FullScan, -Spectral Bkgrnd, Smoothed (0.00,1,GA)
Intens.
[mAU]
x10^4
FCPC_F10_final_RB5_01_33983.d: UV Chromatogram, 200-400 nm
Time [min]

K

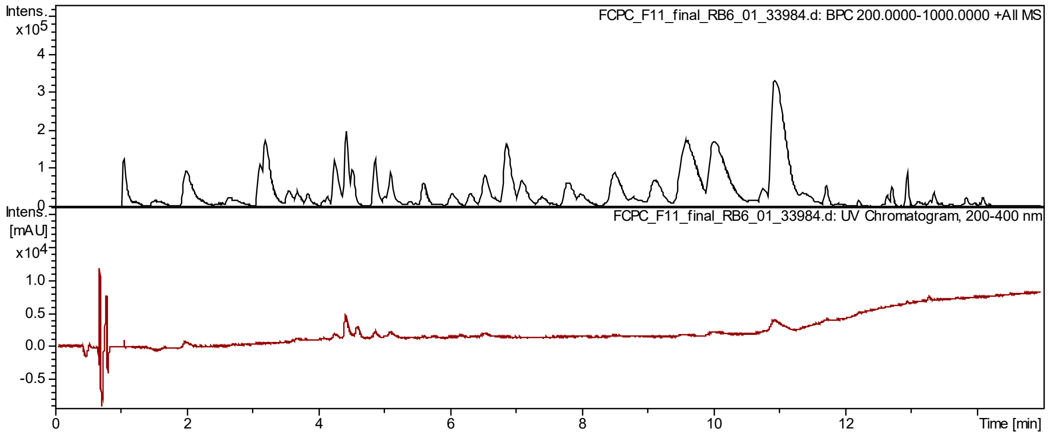
Intens.
x10^5
FCPC_F11_final_RB6_01_33984.d: BPC 200.0000-1000.0000 +All MS
Intens.
[mAU]
x10^4
FCPC_F11_final_RB6_01_33984.d: UV Chromatogram, 200-400 nm
Time [min]

L

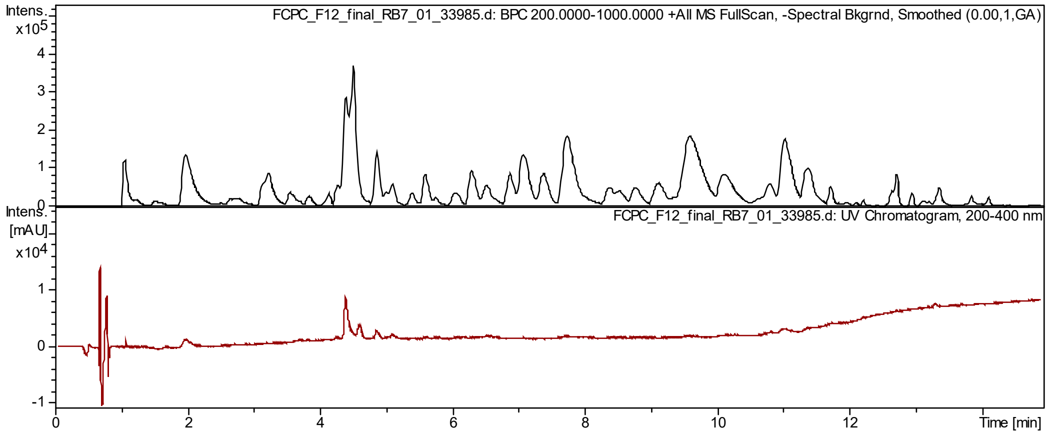
Intens.
x10^5
FCPC_F12_final_RB7_01_33985.d: BPC 200.0000-1000.0000 +All MS FullScan, -Spectral Bkgrnd, Smoothed (0.00,1,GA)
Intens.
[mAU]
x10^4
FCPC_F12_final_RB7_01_33985.d: UV Chromatogram, 200-400 nm
Time [min]

M

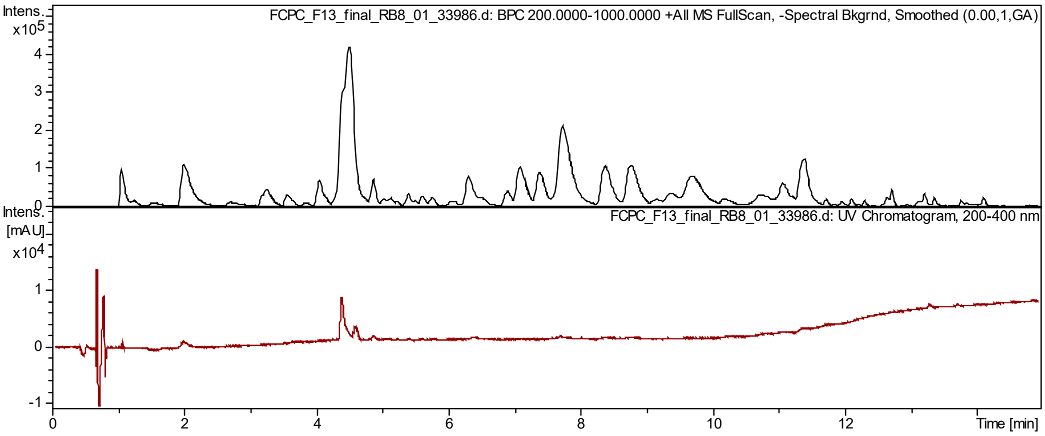

Intens. x10^5
FCPC_F13_final_RB8_01_33986.d: BPC 200.0000-1000.0000 +All MS FullScan, -Spectral Bkgrnd, Smoothed (0.00,1,GA)
Intens. [mAU] x10^4
FCPC_F13_final_RB8_01_33986.d: UV Chromatogram, 200-400 nm
Time [min]

N

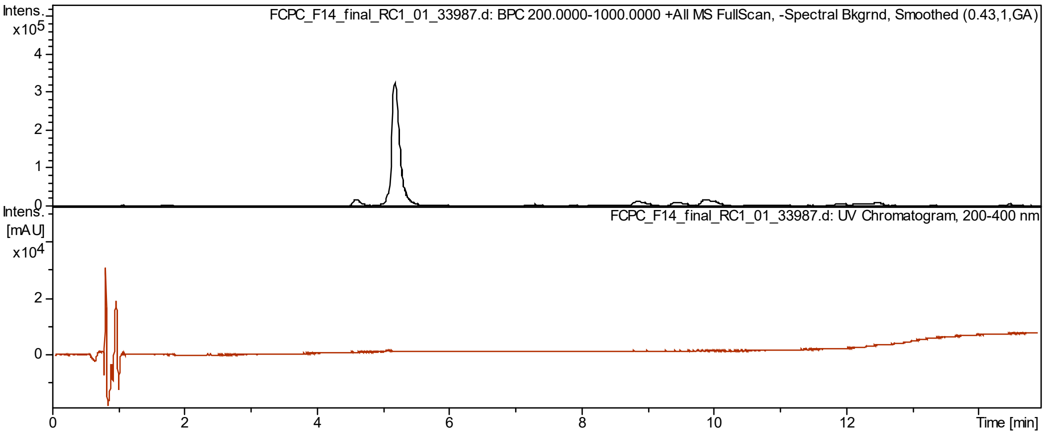

Intens. x10^5
FCPC_F14_final_RC1_01_33987.d: BPC 200.0000-1000.0000 +All MS FullScan, -Spectral Bkgrnd, Smoothed (0.43,1,GA)
Intens. [mAU] x10^4
FCPC_F14_final_RC1_01_33987.d: UV Chromatogram, 200-400 nm
Time [min]

O

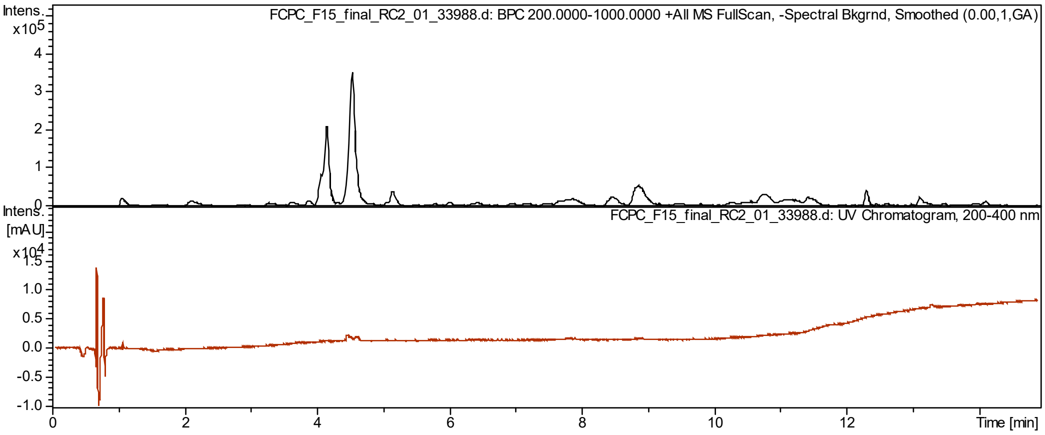

Intens. x10^5
FCPC_F15_final_RC2_01_33988.d: BPC 200.0000-1000.0000 +All MS FullScan, -Spectral Bkgrnd, Smoothed (0.00,1,GA)
Intens. [mAU] x10^4
FCPC_F15_final_RC2_01_33988.d: UV Chromatogram, 200-400 nm
Time [min]

P

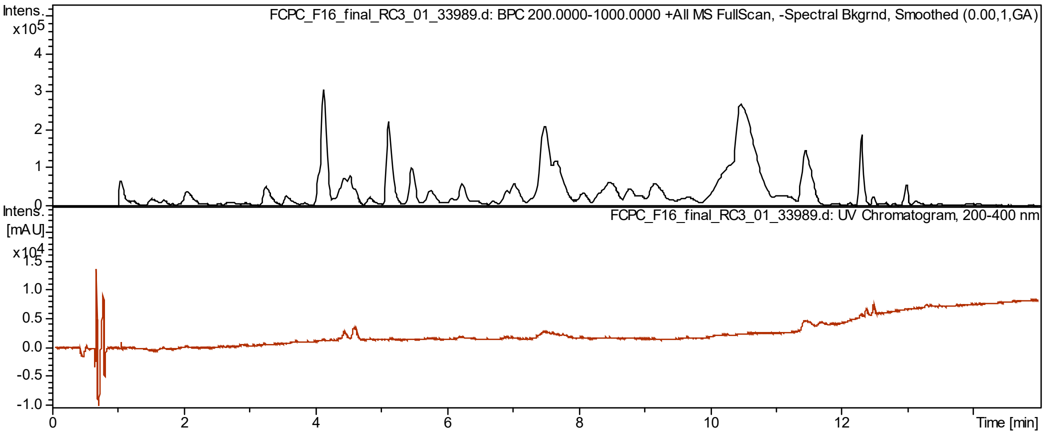
Intens.
x10^5
FCPC_F16_final_RC3_01_33989.d: BPC 200.0000-1000.0000 +All MS FullScan, -Spectral Bkgrnd, Smoothed (0.00,1,GA)
Intens.
[mAU]
x10^4
FCPC_F16_final_RC3_01_33989.d: UV Chromatogram, 200-400 nm
Time [min]

Q

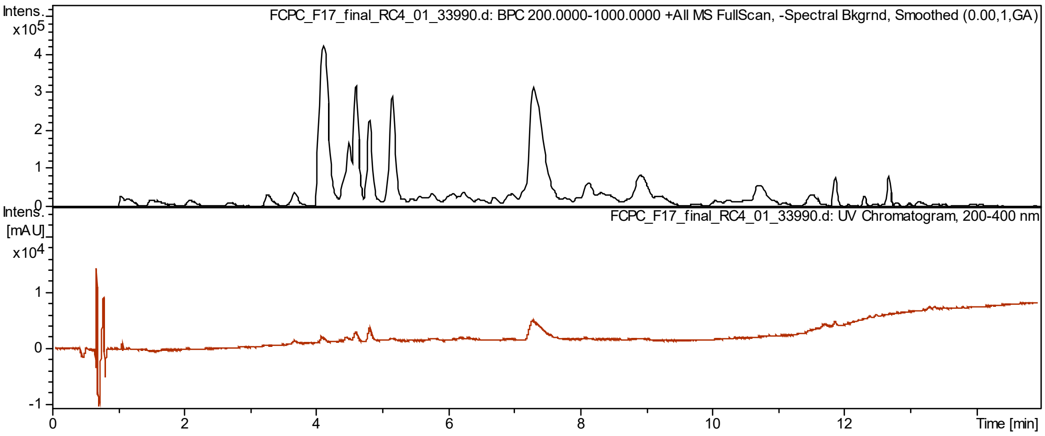
Intens.
x10^5
FCPC_F17_final_RC4_01_33990.d: BPC 200.0000-1000.0000 +All MS FullScan, -Spectral Bkgrnd, Smoothed (0.00,1,GA)
Intens.
[mAU]
x10^4
FCPC_F17_final_RC4_01_33990.d: UV Chromatogram, 200-400 nm
Time [min]

R

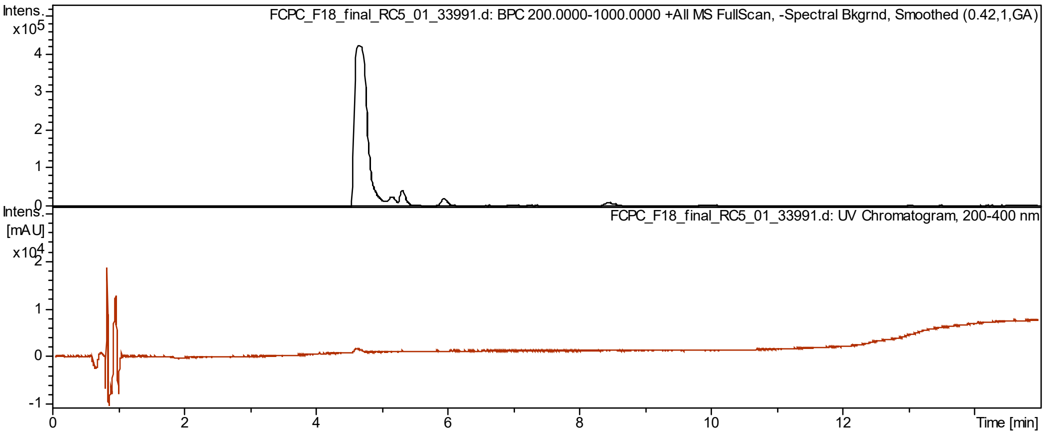
Intens.
x10^5
FCPC_F18_final_RC5_01_33991.d: BPC 200.0000-1000.0000 +All MS FullScan, -Spectral Bkgrnd, Smoothed (0.42,1,GA)
Intens.
[mAU]
x10^4
FCPC_F18_final_RC5_01_33991.d: UV Chromatogram, 200-400 nm
Time [min]

S

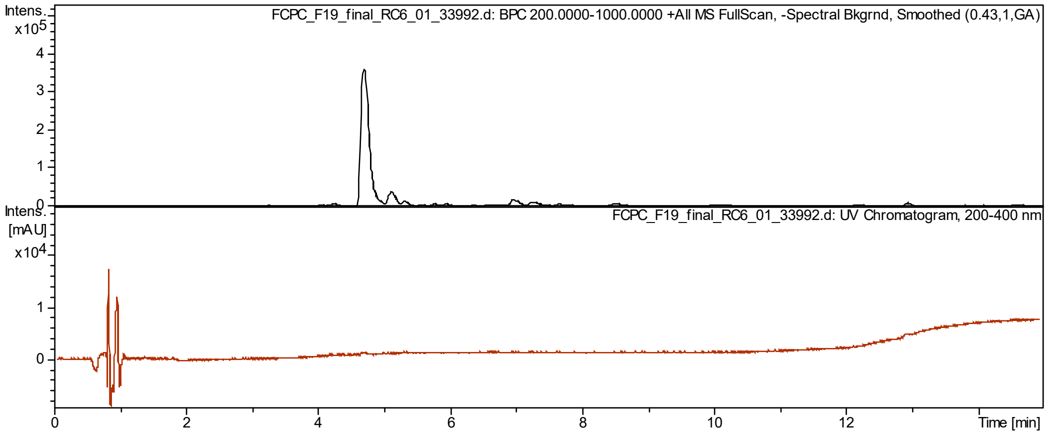

T

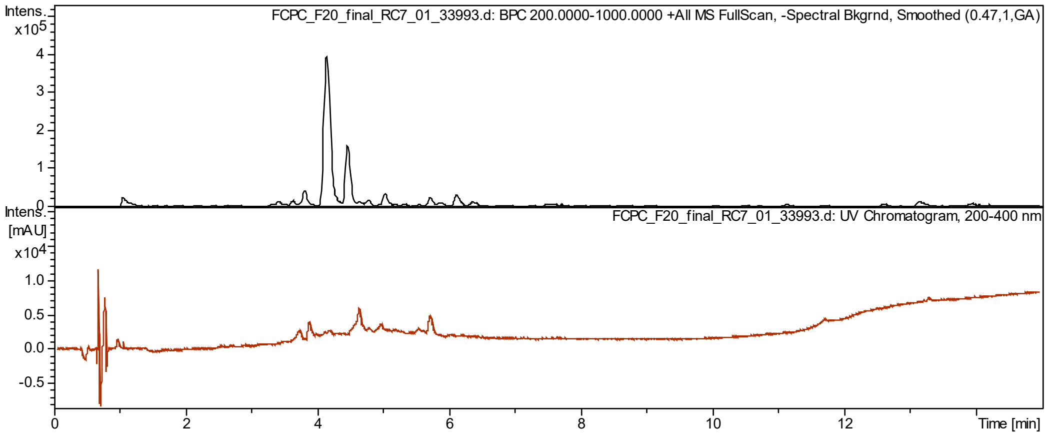

Figure S1. UHPLC/+ESI-QqTOF-MS/MS chromatograms of fractions 1-20. Base peak chromatogram 200.0000-1000.0000 +All MS (black); UV-Chromatogramm, 200-400 nm (red). **A:** Fraction (F) 1. **B**: F2. **C**: F3. **D**: F4. **E**: F5. **F**: F6. **G**: F7. **H**: F8. **I**: F9. **J**: F10. **K**: F11. **L**: F12. **M**: F13. **N**: F14. **O**: F15. **P**: F16. **Q**: F17. **R**: F18. **S**: F19. **T**: F20.

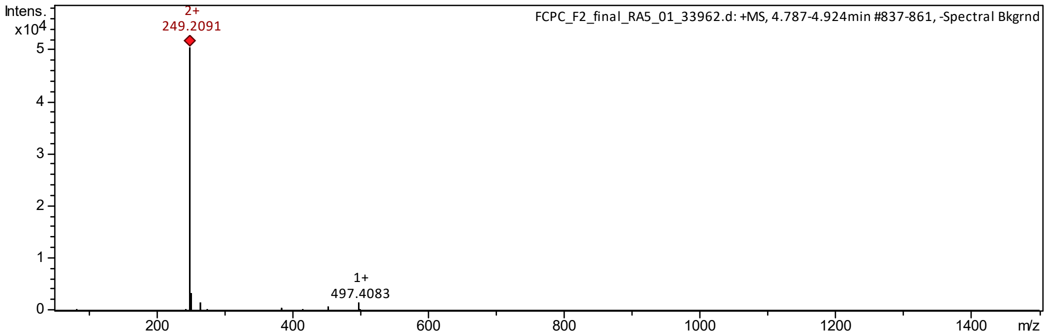

Figure S2. +ESI-QqTOF MS spectrum of compound **1** (O-tigloylcyclovirobuxeine-B [4,9]); m/z 249.2091 $[M+2H]^{2+}$ and 497.4083 $[M+H]^{+}$.

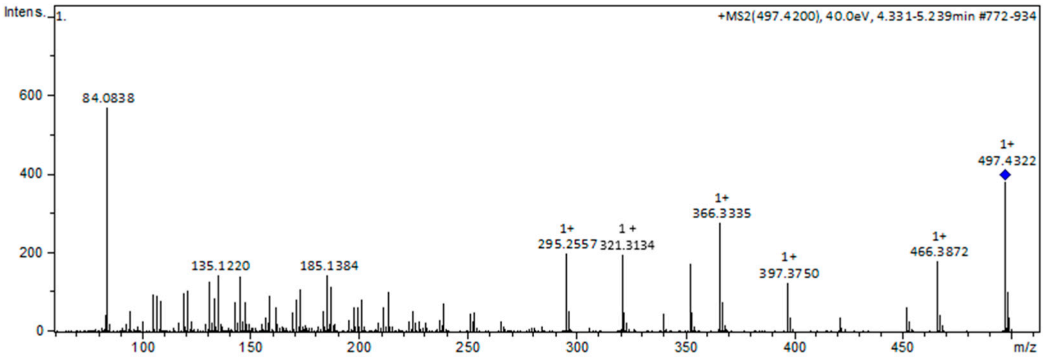

Figure S3. +ESI-QqTOF MS/MS spectrum of compound **1** (O-tigloylcyclovirobuxeine-B [4,9]). The fragmentation pathway was already reported in [9].

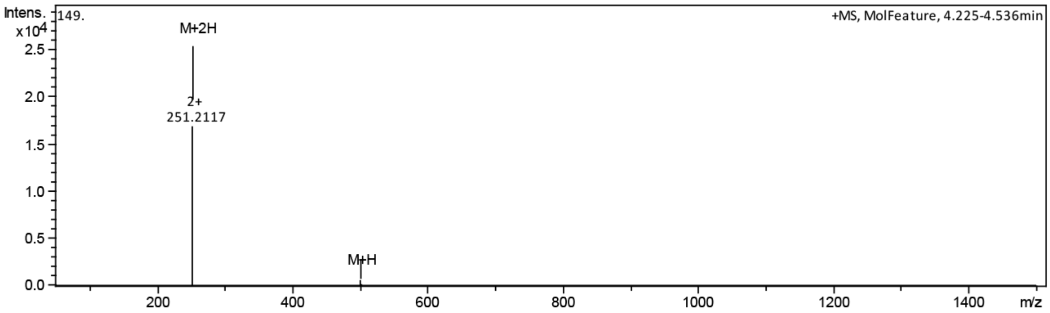

Figure S4. +ESI-QqTOF MS spectrum of compound **2**; m/z 251.2117 $[M+2H]^{2+}$ and 501.4149 $[M+H]^{+}$.

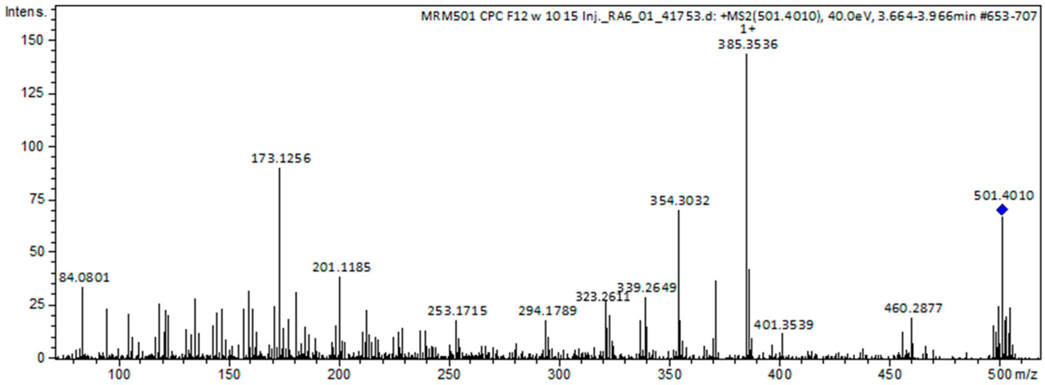

Figure S5. +ESI-QqTOF MS/MS spectrum of compound **2**.

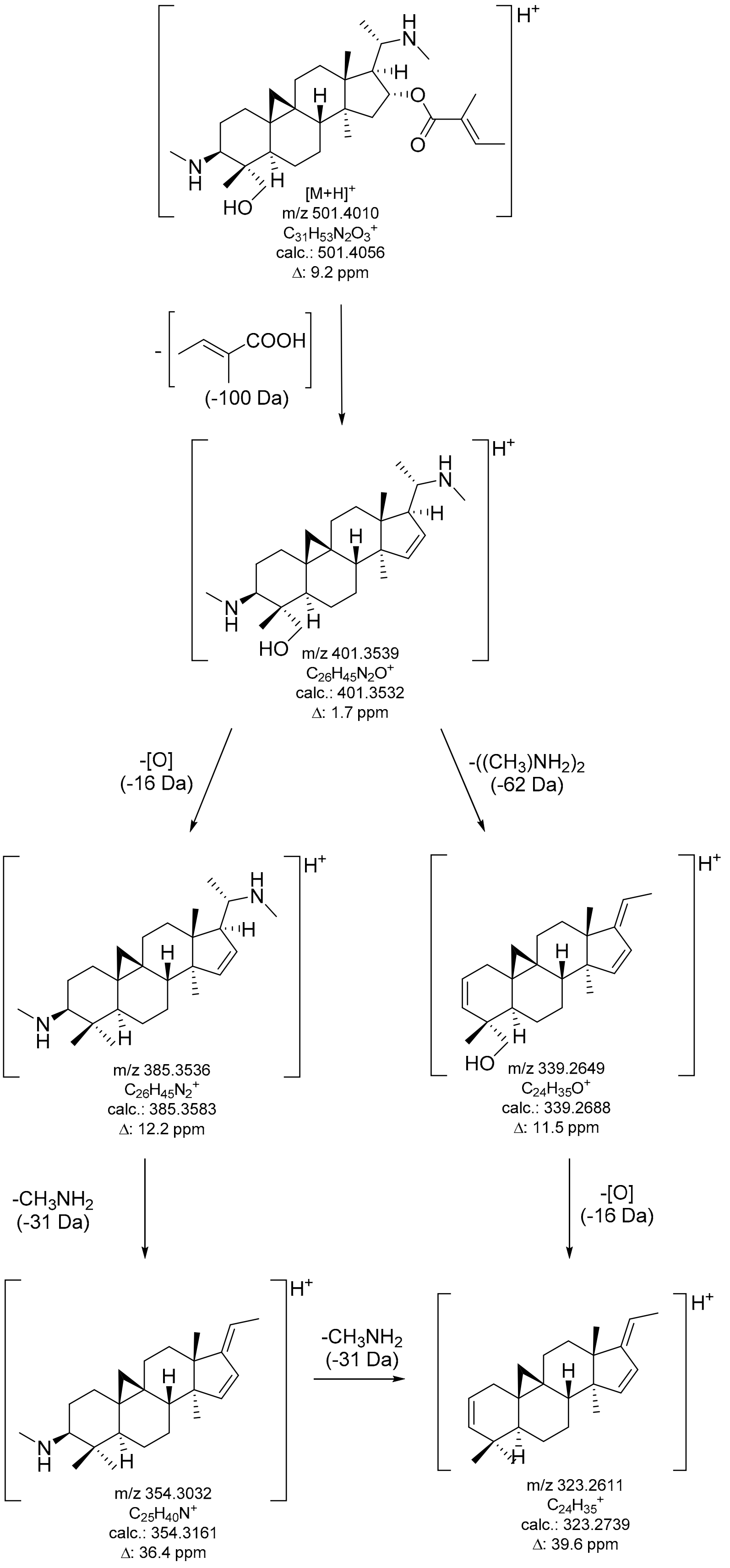

Figure S6. Possible fragmentation pathway of the $[M + H]^+$ ion of compound **2**. (The postulated position of the hydroxyl- and ester group is based on the already known structures of compound **1** and **4**).

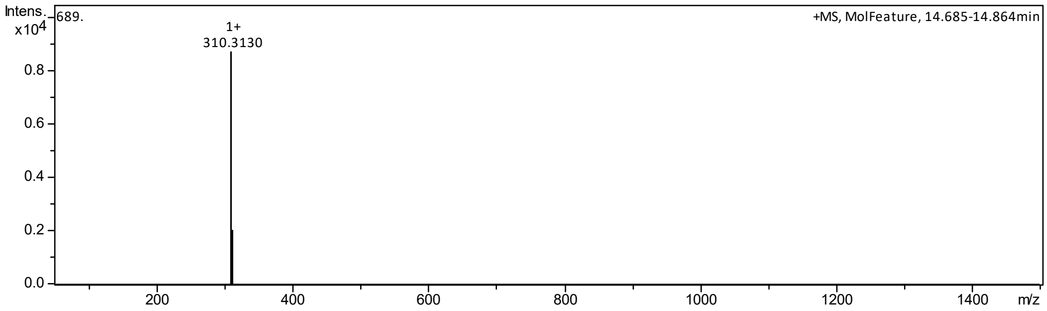

Figure S7. +ESI-QqTOF MS spectrum of compound **3**; 310.3130 [M+H]+.

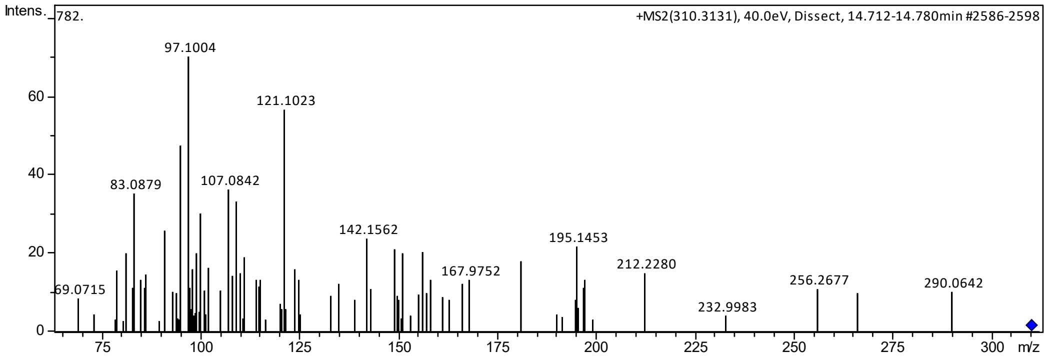

Figure S8. +ESI-QqTOF MS/MS spectrum of compound **3**.

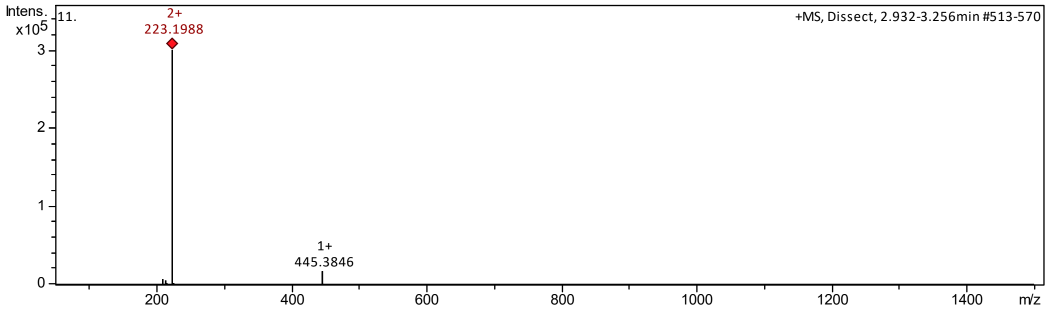

Figure S9. +ESI-QqTOF MS spectrum of compound **4** (cyclomicrophylline-A [4]); m/z 223.1988 $[M+2H]^{2+}$ and 445.3846 $[M+H]^{+}$.

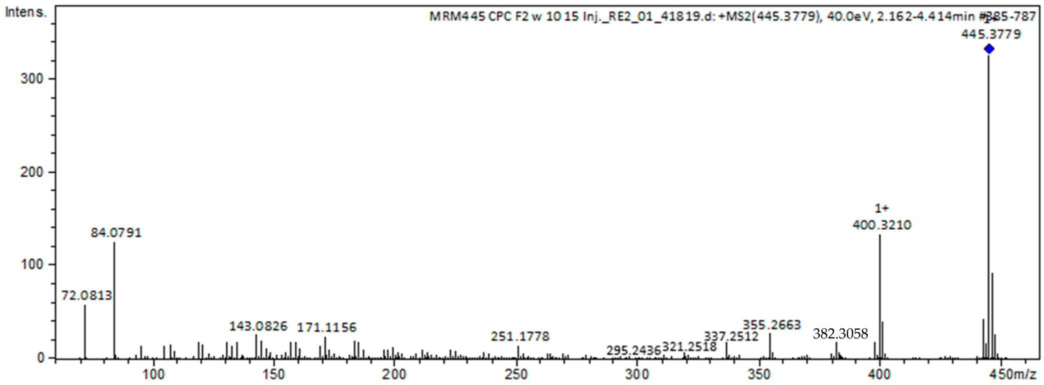

Figure S10. +ESI-QqTOF MS/MS spectrum of compound **4** (cyclomicrophylline-A [4]).

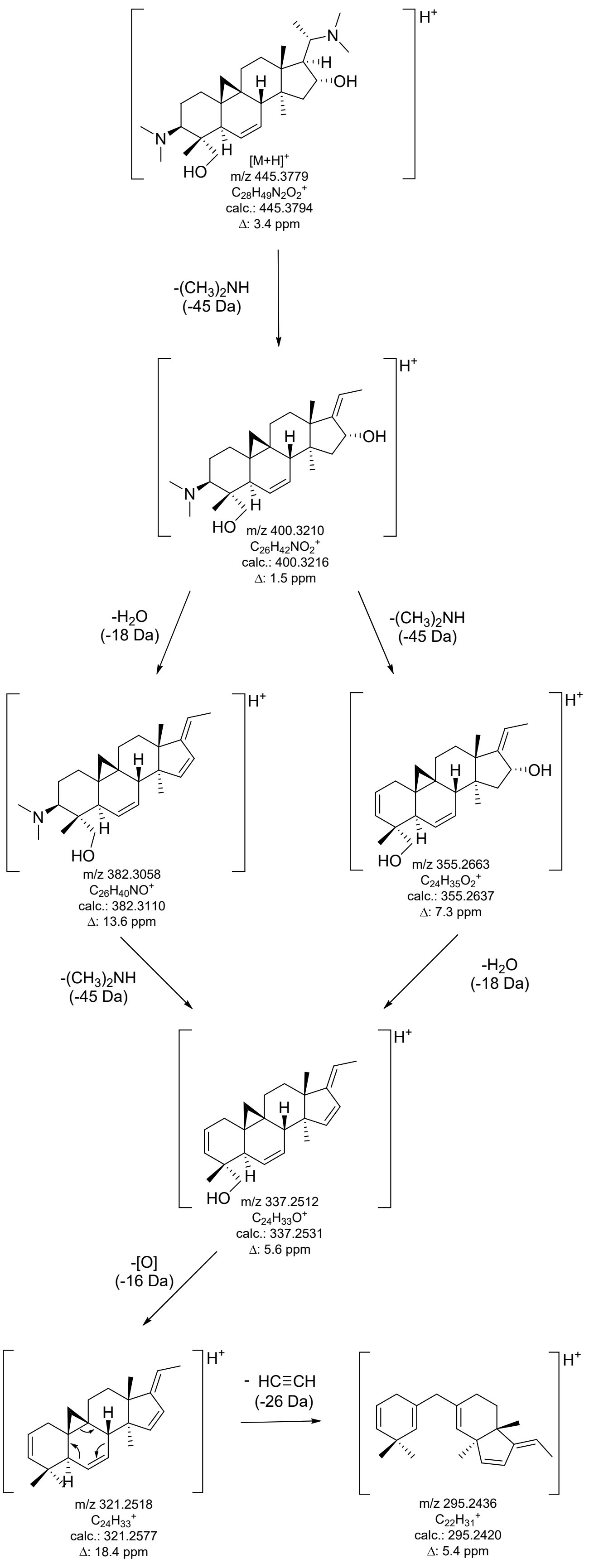

Figure S11. Possible fragmentation pathway of the $[M + H]^+$ ion of compound **4** (cyclomicrophylline-A).

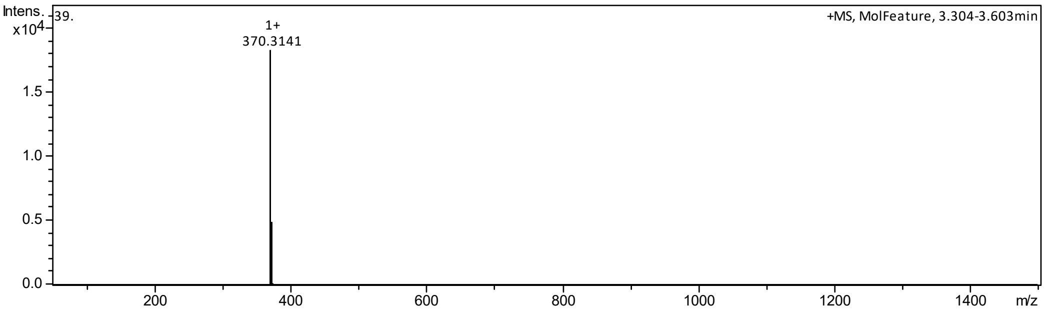

Figure S12. +ESI-QqTOF MS spectrum of compound **5**; 370.3141 [M+H]⁺.

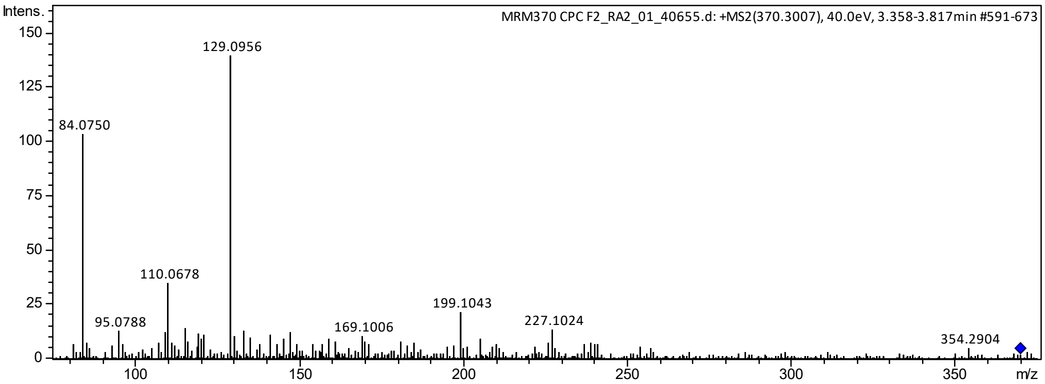

Figure S13. +ESI-QqTOF MS/MS spectrum of compound **5**.

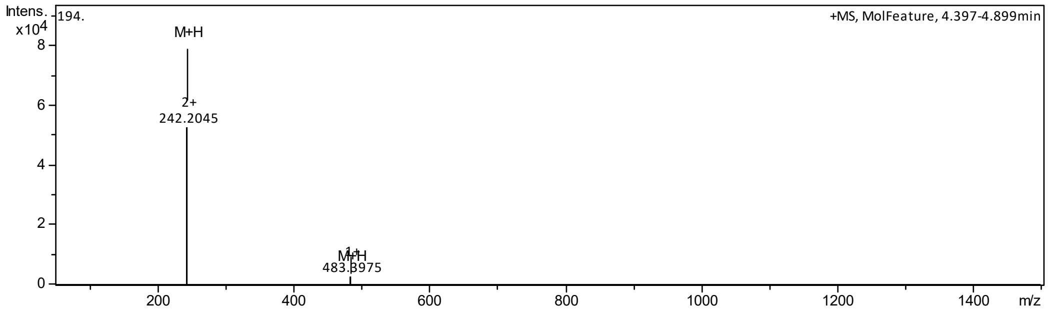

Figure S14. +ESI-QqTOF MS spectrum of compound **6**; m/z 242.2045 $[M+2H]^{2+}$ and 483.3975 $[M+H]^{+}$.

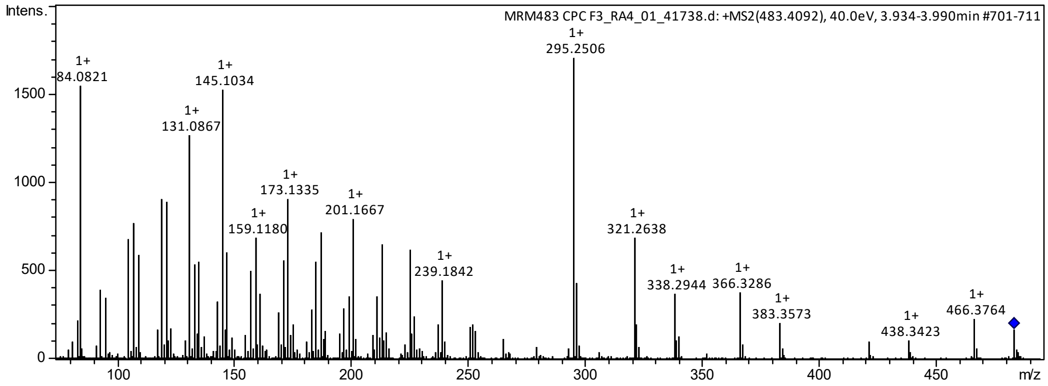

Figure S15. +ESI-QqTOF MS/MS spectrum of compound **6** (detailed in Figure S16).

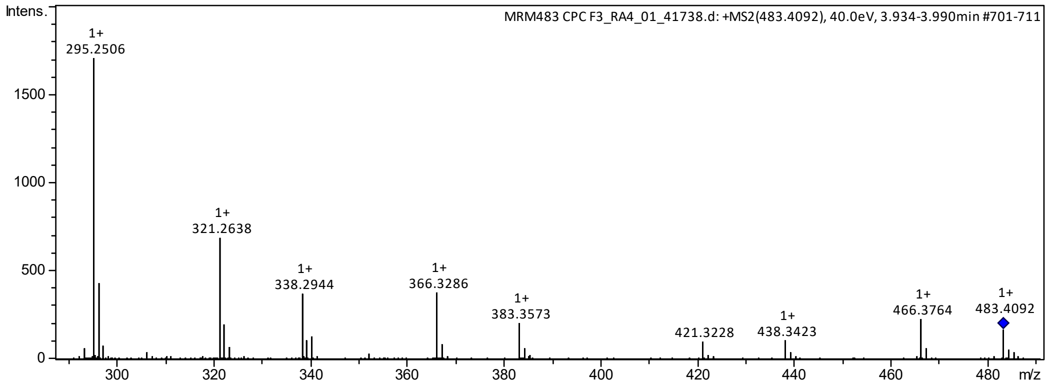

Figure S16. Detailed +ESI-QqTOF MS/MS spectrum of compound **6**.

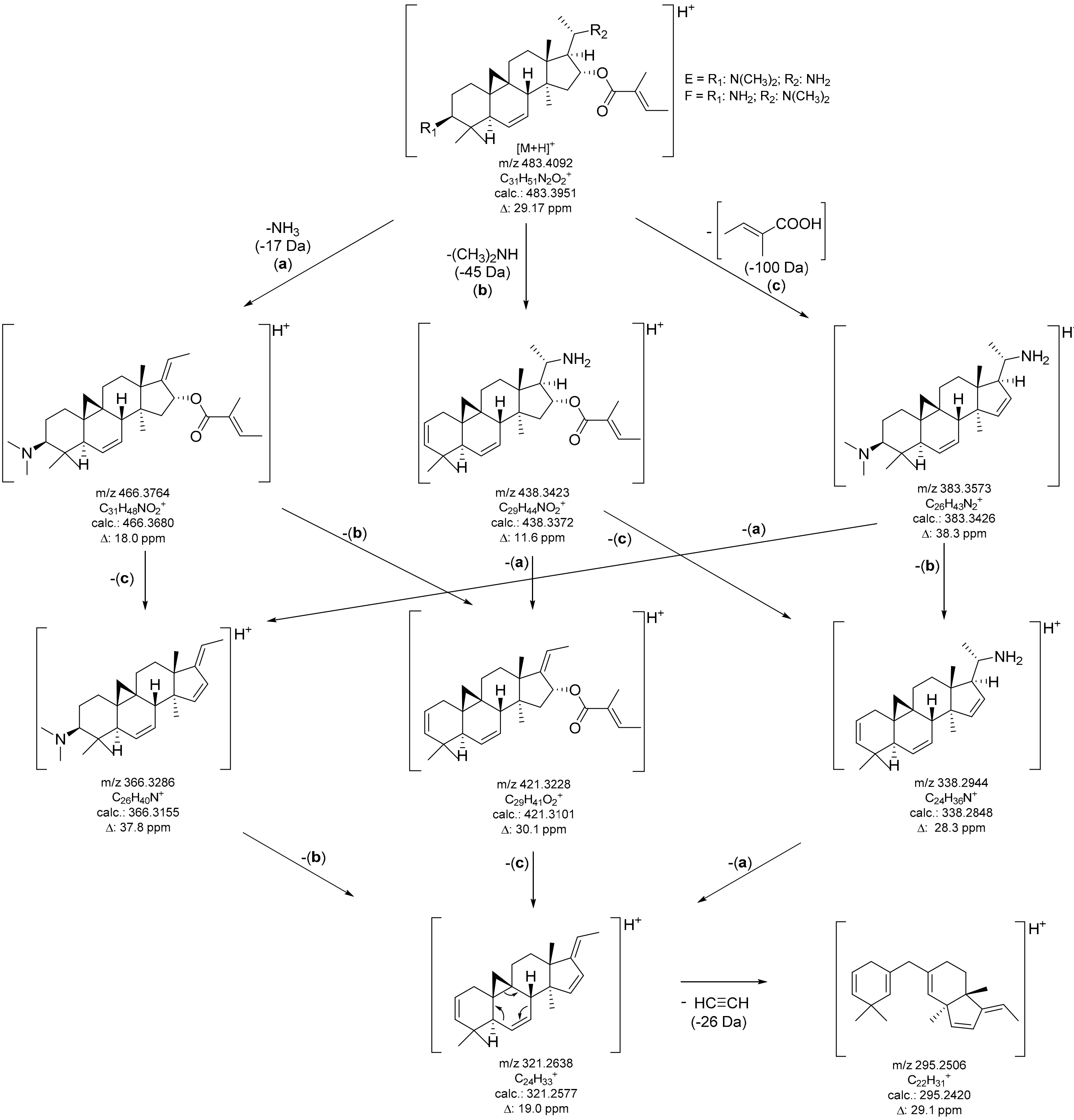

Figure S17. Possible fragmentation pathway of the $[M + H]^+$ ion of compound **6** (O-tigloylcyclovirobuxeine-E or F; fragmentation exemplified for O-tigloylcyclovirobuxeine-E).

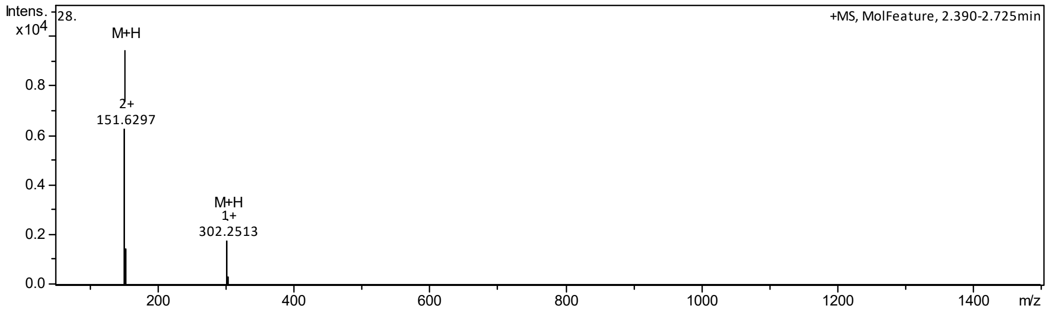

Figure S18. +ESI-QqTOF MS spectrum of compound **7**; m/z 151.6297 $[M+2H]^{2+}$ and 302.2513 $[M+H]^{+}$.

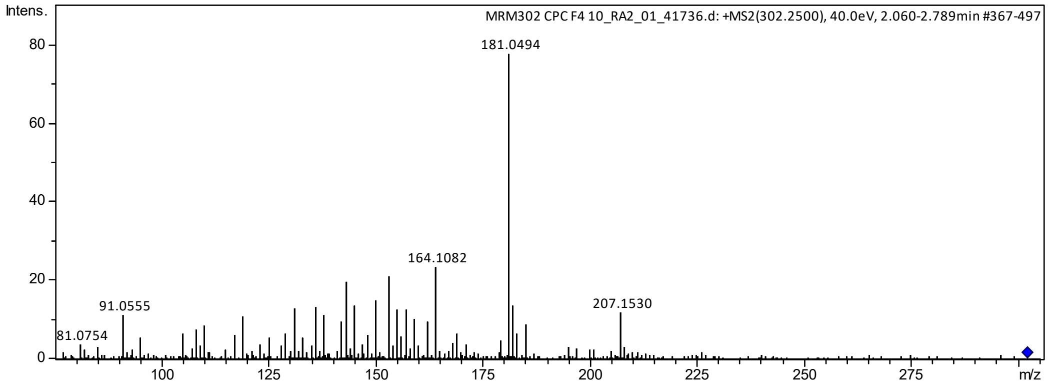

Figure S19. +ESI-QqTOF MS/MS spectrum of compound **7**.

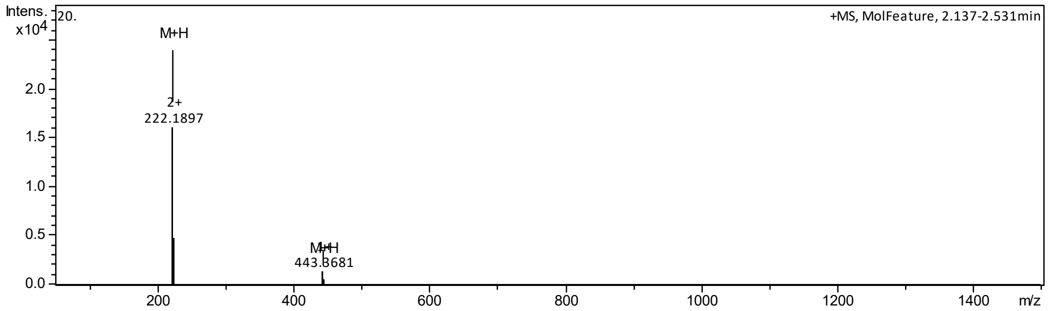

Figure S20. +ESI-QqTOF MS spectrum of compound **8** (N-formylcyclovirobuxeine-B); m/z 222.1897 $[M+2H]^{2+}$ and 443.3681 $[M+H]^{+}$.

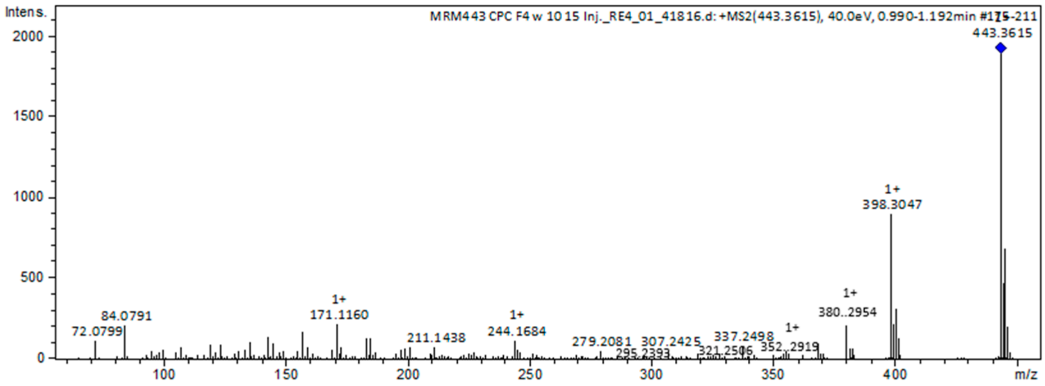

Figure S21. +ESI-QqTOF MS/MS spectrum of compound **8** (N-formylcyclovirobuxeine-B).

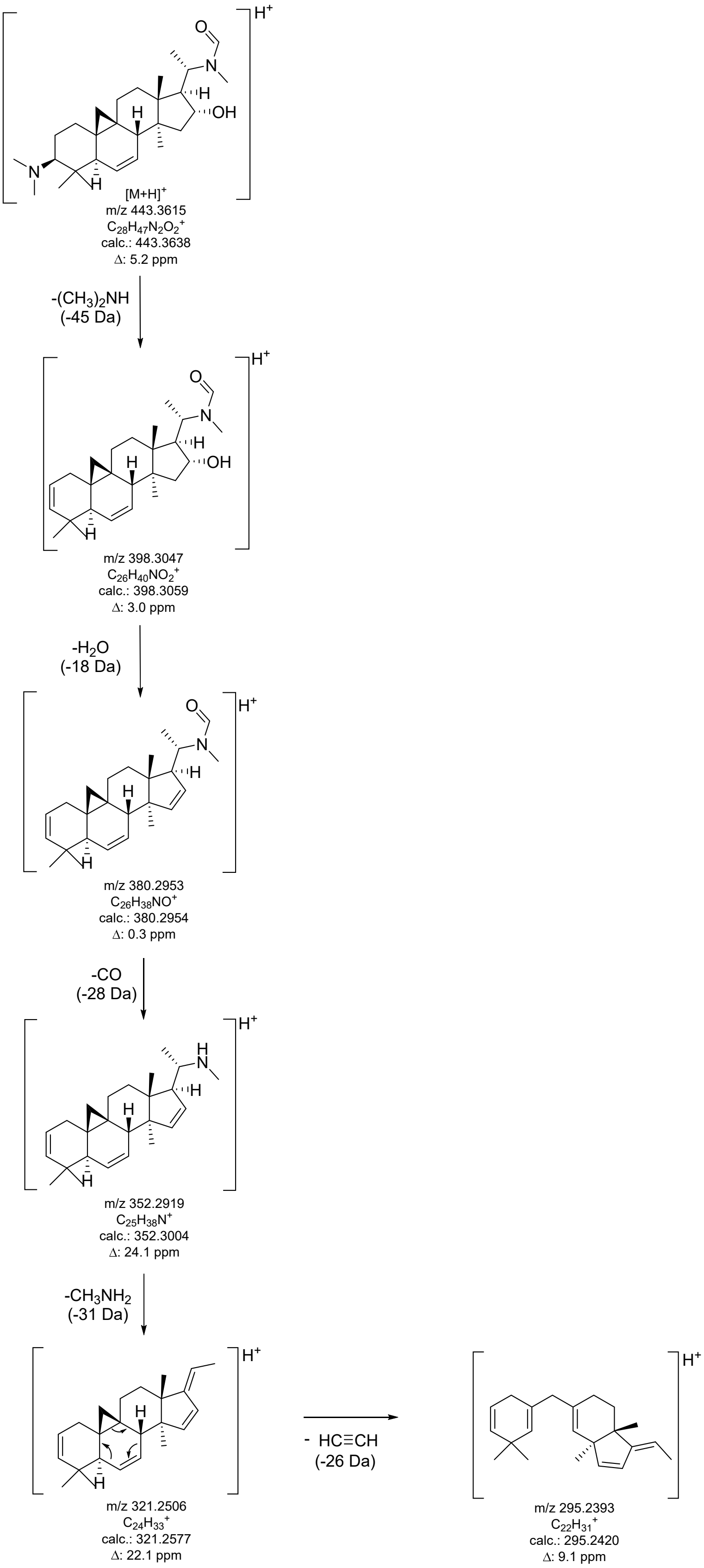

Figure S22. Possible fragmentation pathway of the $[M + H]^+$ ion of compound **8** (N-formylcyclovirobuxeine-B).

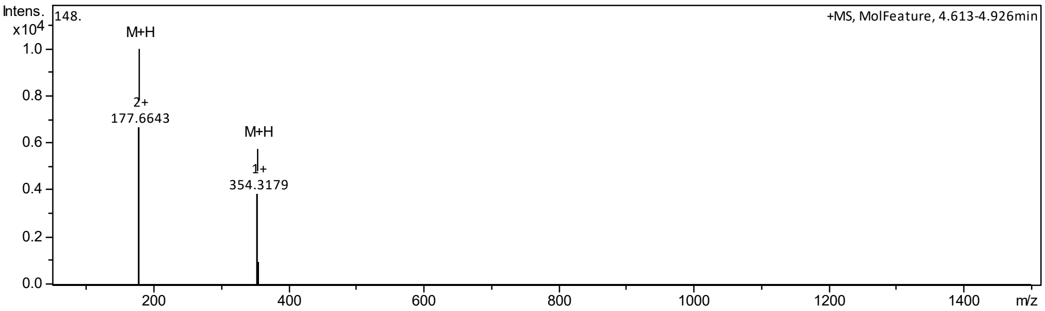

Figure S23. +ESI-QqTOF MS spectrum of compound **9**; m/z 177.6643 $[M+2H]^{2+}$ and 354.3179 $[M+H]^{+}$.

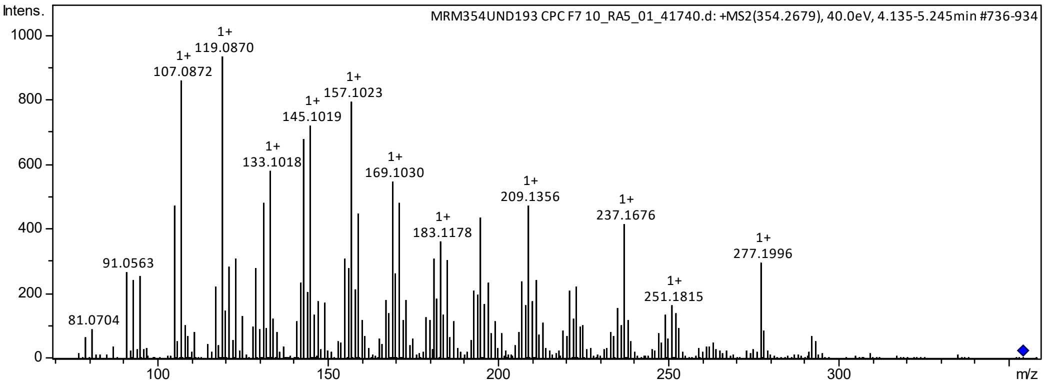

Figure S24. +ESI-QqTOF MS/MS spectrum of compound **9**.

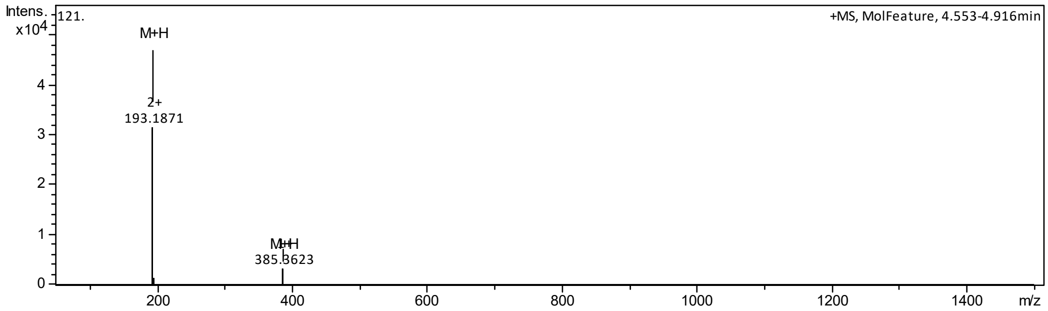

Figure S25. +ESI-QqTOF MS spectrum of compound **10**; m/z 193.1871 $[M+2H]^{2+}$ and 385.3623 $[M+H]^{+}$.

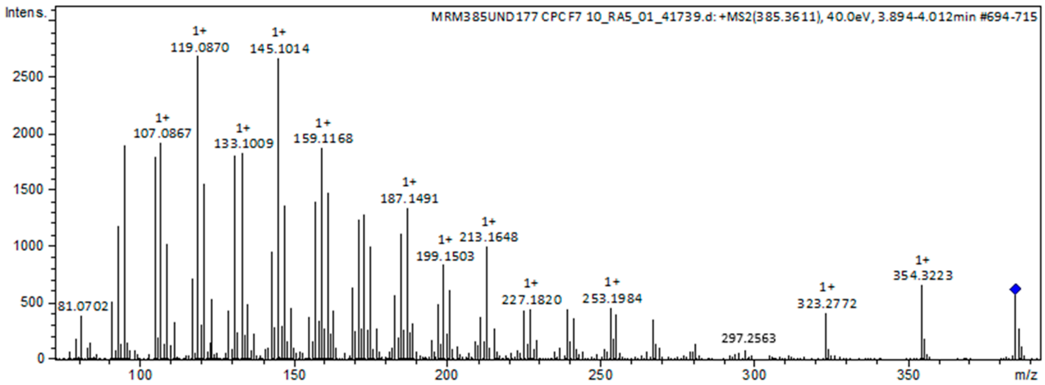

Figure S26. +ESI-QqTOF MS/MS spectrum of compound **10**.

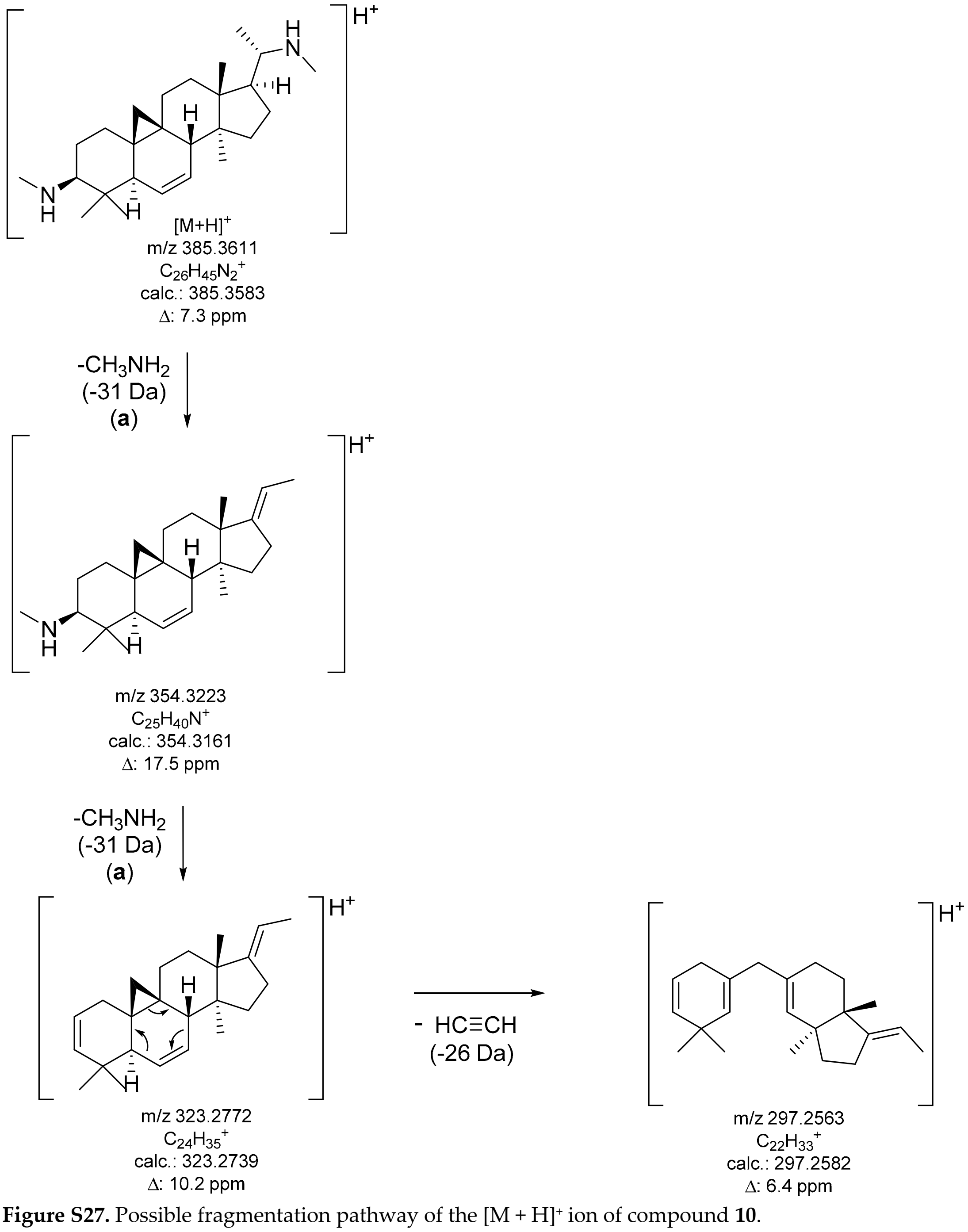

Figure S27. Possible fragmentation pathway of the $[M + H]^+$ ion of compound **10**.

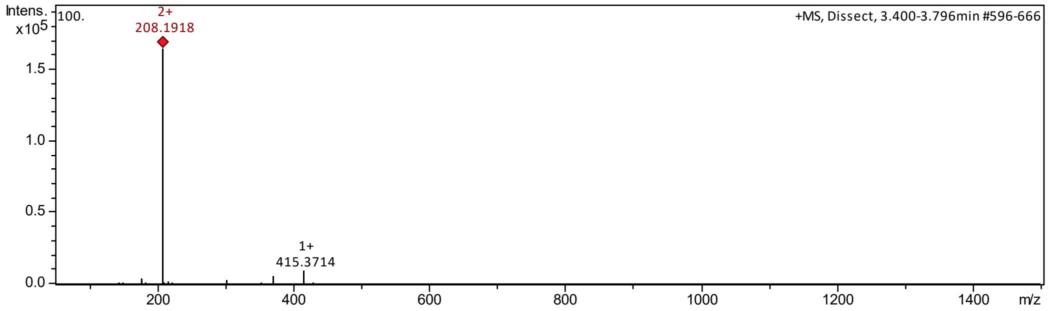

Figure S28. +ESI-QqTOF MS spectrum of compound **11** (cyclovirobuxeine-B [4]); m/z 208.1918 $[M+2H]^{2+}$ and 415.3714 $[M+H]^{+}$.

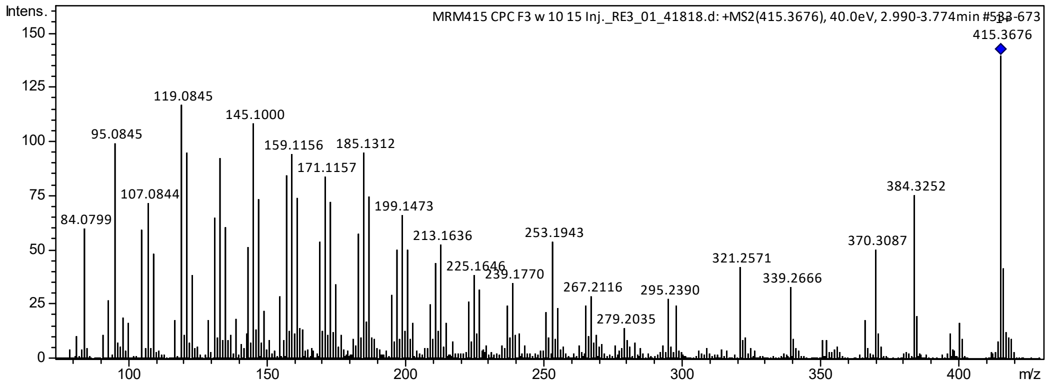

Figure S29. +ESI-QqTOF MS/MS spectrum of compound **11** (cyclovirobuxeine-B [4]).

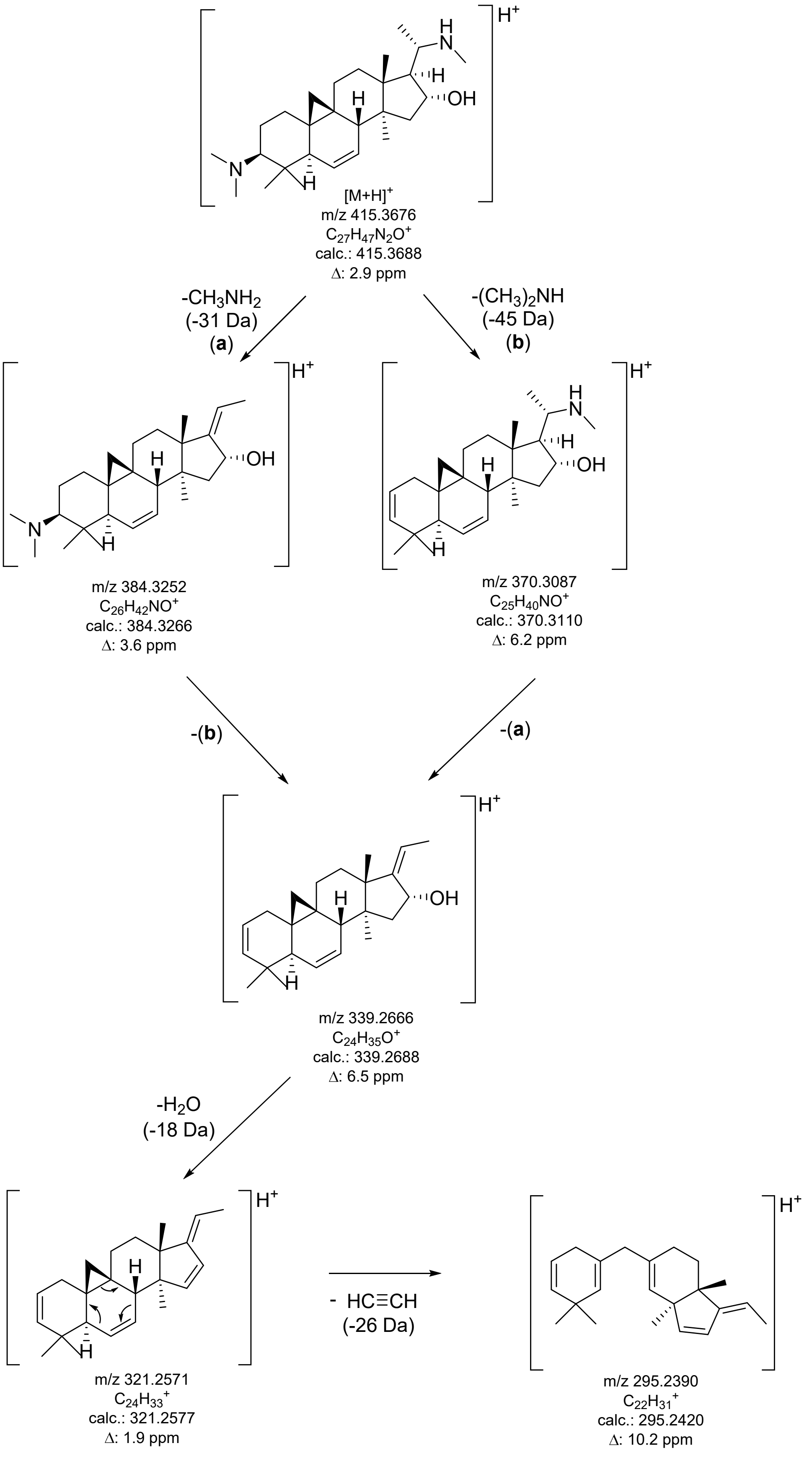

Figure S30. Possible fragmentation pathway of the $[M + H]^+$ ion of compound **11** (cyclovirobuxeine-B).
